# Functional Biomaterials and 3D Bioprinting Approaches for Temporomandibular Joint Reconstruction: A Narrative Review

**DOI:** 10.3390/jfb17080390

**Published:** 2026-08-08

**Authors:** Dobromira Shopova, Svetlin Aleksandrov, Mariya Ivanova Hristozova

**Affiliations:** 1 Department of Prosthetic Dentistry, Faculty of Dental Medicine, Medical University of Plovdiv, 4000 Plovdiv, Bulgaria; svetlin.aleksandrov@mu-plovdiv.bg (S.A.); mariya.hristozova@mu-plovdiv.bg (M.I.H.); 2 CAD/CAM Center, Research Institute, Medical University of Plovdiv, 4000 Plovdiv, Bulgaria

**Keywords:** 3D bioprinting, temporomandibular joint, TMJ regeneration, biomaterials, stem cells, scaffolds

## Abstract

The temporomandibular joint (TMJ) is a highly specialized synovial joint responsible for essential functions such as mastication, speech, and swallowing. Owing to its unique anatomical organization, complex biomechanics, and heterogeneous tissue composition, regeneration of the TMJ remains one of the greatest challenges in craniofacial reconstructive surgery. Conventional treatment modalities, including autologous grafts, alloplastic prostheses, and total joint replacement, are associated with several limitations, including donor-site morbidity, prosthetic wear, limited biological integration, and the inability to restore native tissue architecture. Three-dimensional (3D) bioprinting has emerged as a promising regenerative strategy capable of fabricating patient-specific living constructs that closely mimic the structural and functional characteristics of the native joint. This review summarizes recent advances in TMJ bioprinting, with particular emphasis on the regeneration of the mandibular condylar fibrocartilage, subchondral bone, articular disc, and integrated osteochondral constructs. The literature search covered publications from January 2010 through March 2026, and it was conducted using major scientific databases, including PubMed/MEDLINE, Scopus, Web of Science, and Google Scholar. Current progress in cellular sources, including mesenchymal stem cells and induced pluripotent stem cells, biomaterials and bioinks, growth factor delivery, and multimaterial bioprinting technologies is discussed. Particular attention is given to the challenges associated with reproducing the complex osteochondral interface, achieving adequate vascularization, ensuring long-term mechanical stability, and directing tissue-specific cell differentiation. Emerging technologies, including four-dimensional (4D) bioprinting, decellularized extracellular matrix-based bioinks, artificial intelligence-assisted design, patient-specific computational modeling, and bioreactor-mediated tissue maturation, are highlighted as promising approaches to improve construct functionality and clinical translation. Although the clinical application of TMJ bioprinting remains in its early stages, rapid advances in regenerative medicine and biofabrication technologies indicate that personalized bioengineered joint reconstruction may become a viable therapeutic option for the treatment of severe temporomandibular joint disorders in the future.

## 1. Introduction

The temporomandibular joint is one of the most anatomically and functionally complex synovial joints in the human body. It enables coordinated mandibular movements essential for mastication, speech, swallowing, respiration, and facial expression by integrating rotational (hinge) and translational (sliding) movements within a single bilateral functional unit [1,2]. Unlike most synovial joints, the articular surfaces of the TMJ are covered by dense fibrocartilage rather than hyaline cartilage, providing greater resistance to repetitive mechanical loading and enhanced reparative potential. Another distinctive feature is the presence of a fibrocartilaginous articular disc, which separates the joint into superior and inferior synovial compartments and plays a pivotal role in load distribution, shock absorption, joint lubrication, and maintenance of biomechanical stability [3,4].

The clinical importance of the TMJ is determined not only by its unique anatomy and biomechanics but also by the high prevalence of temporomandibular disorders (TMDs), which represent one of the leading causes of chronic orofacial pain of non-dental origin [5]. Epidemiological studies indicate that signs and symptoms of TMDs affect approximately 5–12% of the adult population, while nearly one-third of adults exhibit at least one clinical sign of TMJ dysfunction. The prevalence is consistently higher among women, particularly between the second and fourth decades of life, suggesting a multifactorial pathogenesis involving biological, biomechanical, hormonal, and psychosocial factors [6].

Temporomandibular disorders comprise a heterogeneous group of musculoskeletal and neuromuscular conditions involving the temporomandibular joints, masticatory muscles, and associated craniofacial structures. Clinically, these disorders are characterized by pain, restricted mandibular movement, joint sounds (clicking, popping, or crepitus), and functional impairment. According to the Diagnostic Criteria for Temporomandibular Disorders (DC/TMD), TMDs are broadly classified into pain-related disorders (including myalgia and arthralgia), intra-articular disorders (primarily disc displacement with or without reduction), degenerative joint diseases such as osteoarthritis, inflammatory arthropathies, traumatic injuries, ankylosis, developmental and congenital abnormalities, and, less frequently, benign or malignant neoplasms affecting the TMJ [7]. Although conservative management remains the first-line treatment for most patients, advanced degenerative or traumatic conditions often result in irreversible damage to the articular cartilage, subchondral bone, and articular disc, highlighting the need for regenerative therapeutic approaches, including tissue engineering and three-dimensional bioprinting [8].

Degenerative and structural disorders of the TMJ, including osteoarthritis, traumatic injuries, ankylosis, neoplastic lesions, and congenital or developmental deformities, may result in progressive joint destruction, severe functional impairment, and craniofacial deformities. Advanced disease is frequently associated with chronic pain, restricted mandibular movement, impaired mastication and speech, reduced maximal mouth opening, and facial asymmetry, all of which substantially compromise patients’ quality of life [9]. In such cases, surgical reconstruction is often required. Conventional reconstructive strategies—including autologous bone and osteochondral grafts, allogeneic grafts, costochondral transplantation, and alloplastic total joint prostheses—have demonstrated clinical utility but remain associated with significant limitations, such as donor-site morbidity, unpredictable graft resorption or overgrowth, limited long-term durability, prosthetic wear, infection, loosening, and the need for revision surgery [10,11].

Because of its unique anatomical organization and functional complexity, the TMJ represents one of the most challenging targets for regenerative medicine and tissue engineering. Unlike many other synovial joints, successful TMJ reconstruction requires the simultaneous regeneration of multiple structurally and functionally distinct tissues, including the fibrocartilaginous articular surface, subchondral bone, and the fibrocartilaginous articular disc, while preserving their biomechanical integration and coordinated function [12,13].

### 1.1. Diseases of the Temporomandibular Joint Suitable for Bioprinting

Not all TMJ disorders represent appropriate indications for tissue engineering and 3D bioprinting. Current regenerative strategies are primarily directed toward pathological conditions characterized by extensive tissue loss or irreversible damage affecting the mandibular condylar cartilage, subchondral bone, or articular disc. The principal objective of these approaches is to restore both the anatomical integrity and biomechanical function of the joint through the fabrication of patient-specific bioengineered constructs capable of replacing or regenerating damaged tissues [14,15].

The classification is presented in Table 1:

### 1.2. Osteoarthritis of the Temporomandibular Joint

Temporomandibular joint osteoarthritis (TMJ-OA) is the most extensively investigated indication for TMJ tissue engineering and bioprinting. It is a chronic degenerative joint disease characterized by progressive degradation of the fibrocartilaginous articular surface, remodeling of the subchondral bone, synovial inflammation, and alterations in the extracellular matrix, ultimately leading to pain, joint dysfunction, and structural deformity [16]. Because the fibrocartilage of the mandibular condyle possesses limited intrinsic regenerative capacity, considerable research has focused on the development of bioprinted osteochondral constructs capable of simultaneously regenerating both cartilaginous and osseous components. Recent advances in biomaterials, stem cell technologies, and multimaterial bioprinting have demonstrated promising results in preclinical models, supporting the potential of personalized osteochondral regeneration for TMJ-OA [17,18].

### 1.3. Severe Condylar Resorption

Progressive condylar resorption is characterized by irreversible loss of condylar bone and cartilage, resulting in decreased ramus height, mandibular retrusion, anterior open bite, facial asymmetry, and temporomandibular joint dysfunction. The condition may occur secondary to autoimmune diseases, inflammatory arthropathies, orthognathic surgery, trauma, or as an idiopathic disorder, particularly in young female patients [19]. Conventional treatment often requires extensive reconstructive procedures using autogenous grafts or alloplastic prostheses. Three-dimensional bioprinting offers the possibility of fabricating patient-specific osteochondral replacements that accurately reproduce the complex geometry of the mandibular condyle while simultaneously promoting biological integration and functional tissue regeneration [20,21].

### 1.4. Temporomandibular Joint Ankylosis

Temporomandibular joint ankylosis is a debilitating condition characterized by fibrous or bony fusion between the mandibular condyle and the temporal bone, leading to partial or complete loss of joint mobility. Patients typically present with severe limitation of mouth opening, impaired mastication and speech, facial asymmetry, and, in growing individuals, significant craniofacial developmental disturbances [22]. Surgical gap arthroplasty, interpositional arthroplasty, and total joint reconstruction remain the current standards of care; however, recurrence, heterotopic bone formation, donor-site morbidity, and prosthetic complications continue to limit long-term outcomes. Tissue-engineered and bioprinted osteochondral constructs have therefore emerged as promising alternatives, with the potential to restore joint morphology and function while reducing the risk of re-ankylosis through biological regeneration of native tissues [17,23,24].

### 1.5. Traumatic Condylar Defects

Traumatic injuries of the mandibular condyle, particularly displaced condylar fractures and high-energy craniofacial trauma, may result in substantial loss of both osseous and fibrocartilaginous tissues. Extensive defects often require complex reconstructive procedures involving autologous bone grafts, vascularized free flaps, or alloplastic joint prostheses. However, these approaches are associated with donor-site morbidity, limited anatomical conformity, and variable long-term functional outcomes [25]. Three-dimensional bioprinting offers the possibility of fabricating patient-specific osteochondral constructs that accurately reproduce the external morphology and internal architecture of the mandibular condyle while promoting biological integration and tissue regeneration [15,17,26].

### 1.6. Degeneration and Perforation of the Articular Disc

The temporomandibular joint articular disc is one of the most challenging structures to regenerate because of its highly specialized fibrocartilaginous composition, anisotropic collagen organization, and limited intrinsic healing capacity. Disc degeneration, perforation, and irreducible disc displacement are among the principal targets of current tissue engineering strategies. Recent studies have focused on the development of multilayer bioprinted scaffolds incorporating fibrochondrocytes, mesenchymal stem cells, and bioactive hydrogels to reproduce the zonal organization and biomechanical properties of the native disc [27,28]. These approaches aim to restore both the structural integrity and functional performance of the articular disc while minimizing the risk of recurrent degeneration [15].

### 1.7. Congenital and Acquired Condylar Defects

Congenital abnormalities of the mandibular condyle, including condylar aplasia, hypoplasia, and craniofacial malformations such as hemifacial microsomia, as well as acquired defects following trauma, infection, or surgical resection, represent major reconstructive challenges. Conventional treatment often requires staged surgical procedures and may fail to achieve complete restoration of joint morphology and function [25]. Patient-specific bioprinted osteochondral constructs have the potential to provide anatomically accurate replacements capable of integrating with surrounding tissues and supporting physiological remodeling. In pediatric patients, future bioengineered constructs may also offer the possibility of accommodating craniofacial growth, although this concept remains experimental [29,30].

### 1.8. Post-Resection Defects Following Tumor Surgery

Surgical treatment of benign and malignant tumors involving the mandibular condyle or temporomandibular joint frequently results in extensive composite defects affecting bone, cartilage, and surrounding soft tissues [31]. Reconstruction of these defects remains technically demanding and often requires multiple surgical procedures. Advances in computer-assisted design (CAD), medical imaging, and additive manufacturing enable the generation of patient-specific bioprinted constructs based on computed tomography (CT), cone-beam computed tomography (CBCT), or magnetic resonance imaging (MRI) datasets. Such individualized constructs have the potential to improve anatomical accuracy, functional rehabilitation, and long-term integration with host tissues [15,17,32].

Collectively, these pathological conditions are characterized by irreversible destruction or loss of one or more essential components of the temporomandibular joint, thereby creating a strong rationale for regenerative therapeutic strategies. Current research in TMJ bioprinting is primarily focused on the fabrication of integrated osteochondral constructs capable of simultaneously regenerating the mandibular condylar fibrocartilage, subchondral bone, and fibrocartilaginous articular disc—the three principal tissues responsible for joint biomechanics and function. The convergence of stem cell biology, biomaterial science, three-dimensional bioprinting, and computational design is expected to facilitate the development of biologically functional, patient-specific TMJ replacements that may ultimately overcome many of the limitations associated with conventional reconstructive techniques.

Although several reviews have discussed tissue engineering or regenerative approaches for the TMJ, most focus primarily on biomaterials, scaffold fabrication, or general tissue engineering principles. Few reviews systematically integrate tissue-specific bioprinting strategies for the individual TMJ components while simultaneously addressing their clinical indications, biological requirements, and translational challenges. The present review’s targets are to fill this gap by providing a comprehensive tissue-oriented analysis together with a critical evaluation of emerging technologies that may facilitate future clinical application.

The aim of this narrative review is to evaluate current advances in three-dimensional (3D) bioprinting for TMJ reconstruction, with particular emphasis on tissue-specific regenerative strategies for condylar cartilage, subchondral bone, the articular disc, and osteochondral interfaces. Specifically, this review addresses the following questions:

1. To what extent can current 3D bioprinting technologies reproduce the complex architecture and function of the multiple tissues composing the TMJ?
2. Which cellular sources, biomaterials, bioinks, and biofabrication strategies show the greatest potential for clinically relevant TMJ regeneration?
3. What are the major biological, mechanical, and translational challenges that currently limit clinical implementation?
4. Which emerging technologies are most likely to facilitate future clinical translation?

## 2. Materials and Methods

### 2.1. Study Design

This study was designed as a narrative literature review focusing on advances in 3D bioprinting approaches for TMJ reconstruction, including the regeneration of condylar fibrocartilage, subchondral bone, articular disc, and osteochondral interfaces. The aim was to critically synthesize current knowledge on cellular sources, biomaterials, biofabrication strategies, and emerging technologies relevant to TMJ tissue engineering and regenerative medicine.

### 2.2. Literature Search Strategy

The literature search covered publications from January 2010 through March 2026. A comprehensive literature search was conducted using major scientific databases, including PubMed/MEDLINE, Scopus, Web of Science, and Google Scholar. The search was performed using combinations of the following keywords: temporomandibular joint, TMJ, bioprinting, 3D bioprinting, tissue engineering, osteochondral regeneration, fibrocartilage, subchondral bone, articular disc, bioink, mesenchymal stem cells, and regenerative medicine. Additional relevant articles were identified through manual screening of reference lists from selected publications and review articles to ensure inclusion of seminal and highly cited studies. The literature search strategy in detail is presented in Table 2.

### 2.3. Inclusion and Exclusion Criteria

Included studies met the following criteria: published in peer-reviewed scientific journals, written in English, focused on TMJ biology, pathology, or regenerative approaches, addressed 3D bioprinting, tissue engineering, biomaterials, or related cellular and molecular strategies applicable to osteochondral or fibrocartilaginous regeneration. Exclusion criteria were non-peer-reviewed publications (unless highly relevant foundational reports), studies unrelated to craniofacial or osteochondral tissues, purely clinical reports without relevance to regenerative or bioengineering approaches.

### 2.4. Data Extraction and Analysis

A total of 427 publications were initially identified. After title, abstract and full-text screening according to the inclusion criteria, 100 publications were included in the final qualitative synthesis. Relevant information was extracted and organized into thematic categories, including:

1. TMJ anatomy and pathology relevant to regeneration;
2. Cellular sources for fibrocartilage and bone engineering;
3. Biomaterials and bioink development;
4. Growth factors and signaling pathways;
5. Bioprinting technologies (single-material, multimaterial, and 4D bioprinting);
6. Osteochondral construct design and interface engineering;
7. Emerging technologies (AI, computational modeling, bioreactors, vascularization strategies).

The literature was synthesized qualitatively, with emphasis on identifying current trends, limitations, and translational challenges in TMJ bioprinting.

## 3. Results

**Figure 1:**
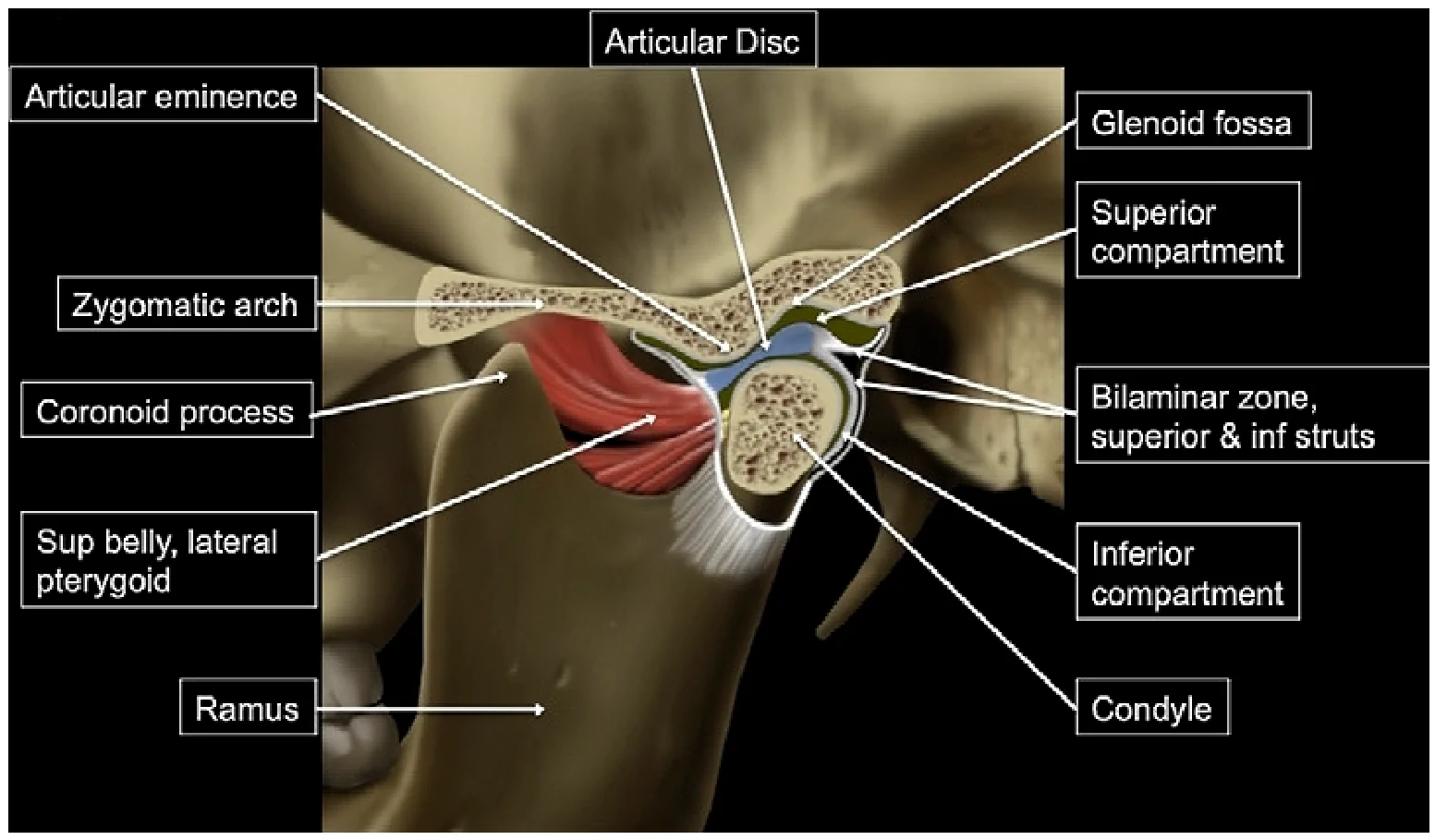
Anatomical features of the TMJ. Reprinted from Ref. [33].

**Figure 2:**
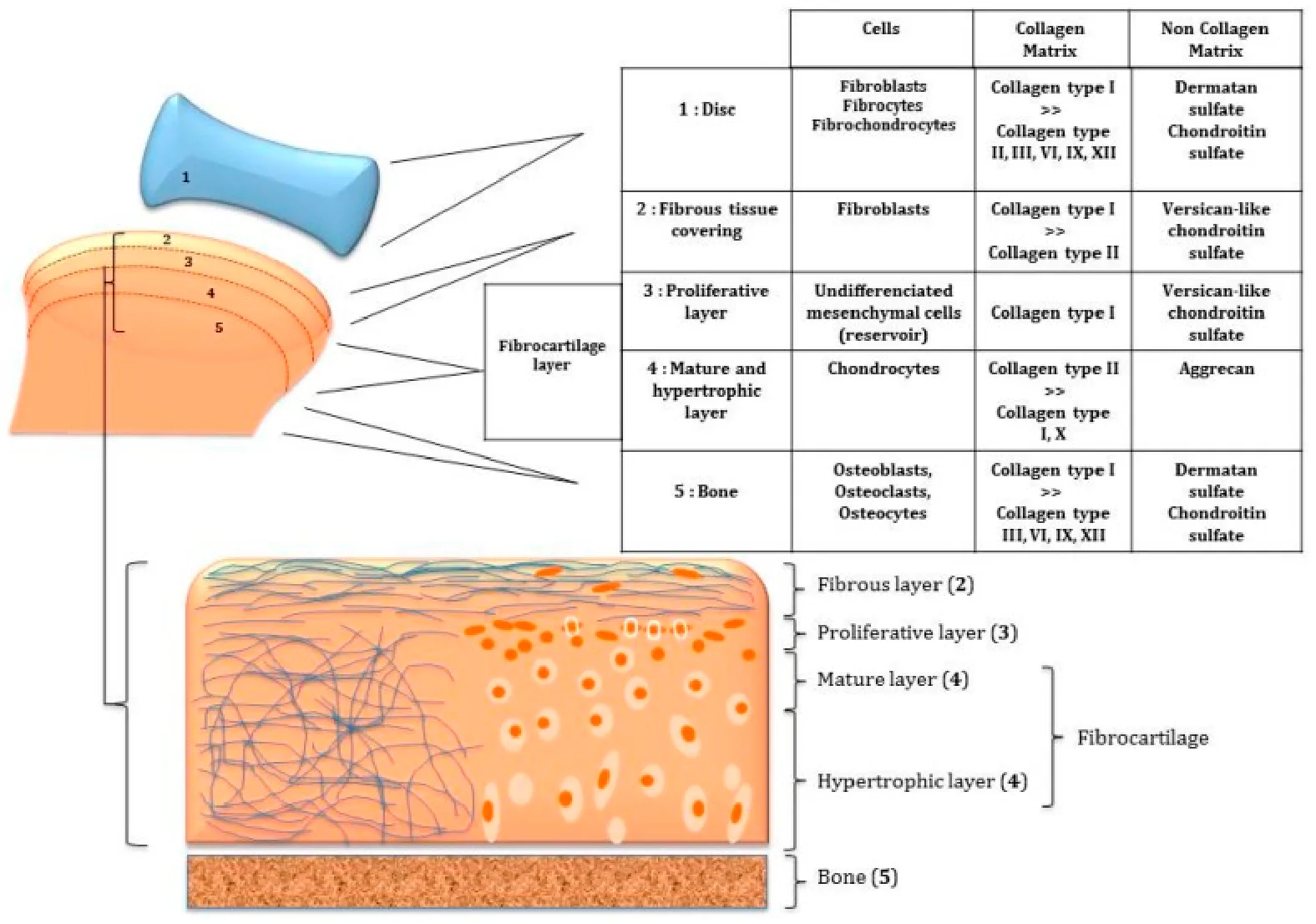
Scheme of the composition of the five compartments of TMJ to regenerate. Reprinted from Ref. [34].

The regeneration of the temporomandibular joint requires a tissue-specific approach because each anatomical component exhibits distinct structural, biological, and biomechanical characteristics (Figure 1) [33]. Consequently, current bioprinting strategies are not directed toward reconstruction of the entire joint as a single unit but rather toward the regeneration of its principal functional components, including the condylar fibrocartilage, subchondral bone, articular disc, and integrated osteochondral interface. Each tissue requires specific cellular sources, biomaterials, bioinks, and bioactive molecules to reproduce its native architecture and function (Figure 2) [34]. The following sections summarize the current state of knowledge regarding tissue-specific bioprinting strategies and their potential clinical applications.

### 3.1. Bioprinting of Condylar Cartilage

Conventional surgical approaches, including autologous grafting and alloplastic TMJ prostheses, are limited in their ability to fully restore the native biological structure, zonal organization, and biomechanical function of condylar cartilage. These limitations have driven increasing scientific interest toward 3D bioprinting as a strategy for the fabrication of living tissue constructs capable of recapitulating both the architecture and functional behavior of the mandibular condylar surface [35,36].

The condylar cartilage of the TMJ represents a specialized form of secondary fibrocartilage, which differs fundamentally from hyaline articular cartilage found in other synovial joints. It is predominantly composed of dense fibrous connective tissue enriched with type I collagen, with a lower proportion of type II collagen, which contributes to its unique ability to withstand combined compressive, tensile, and shear loading conditions characteristic of mandibular function [37]. However, due to its limited vascularization, low cellular turnover, and reduced intrinsic regenerative capacity, condylar cartilage exhibits poor healing potential following trauma, degenerative changes, or inflammatory joint diseases [38,39].

In this context, bioprinting-based cartilage engineering enables precise spatial deposition of cells, biomaterials, and bioactive factors, thereby creating controlled microenvironments that support chondrogenic differentiation and extracellular matrix formation. Mesenchymal stem cells (MSCs), fibrochondrocytes, and bioactive hydrogels can be organized in a zonal architecture that more closely mimics native condylar cartilage structure and function [35,37,40].

Cellular Sources

The success of TMJ cartilage bioprinting is highly dependent on the selection of appropriate cellular sources. The most commonly utilized cell types include bone marrow-derived mesenchymal stem cells (BM-MSCs), adipose-derived mesenchymal stem cells (AD-MSCs), dental pulp stem cells (DPSCs), induced pluripotent stem cells (iPSCs), chondrocytes, and fibrochondrocytes. Among these, mesenchymal stem cells are widely regarded as the preferred cell source due to their high proliferative capacity and their ability to undergo chondrogenic and osteogenic differentiation under defined biochemical and mechanical stimulation. This plasticity makes MSCs particularly suitable for the fabrication of multiphasic osteochondral constructs relevant to temporomandibular joint regeneration [41,42,43].

Biomaterials and Bioinks

Biomaterials are natural or synthetic materials intended to interact with biological tissues and provide structural or biological support for tissue regeneration. Bioinks, in contrast, are printable formulations composed of biomaterials combined with living cells and/or bioactive molecules that are specifically designed for three-dimensional bioprinting. Thus, while all bioinks contain biomaterials, not all biomaterials are suitable for use as bioinks because bioinks must additionally possess appropriate rheological properties, printability, and cytocompatibility [44,45]. Bioinks used for condylar cartilage bioprinting must simultaneously ensure high cell viability, suitable rheological properties for printing, and adequate mechanical stability after fabrication. A variety of natural and synthetic biomaterials have been investigated, including collagen, gelatin methacrylate (GelMA), alginate, hyaluronic acid, fibrin, polyethylene glycol (PEG), and polycaprolactone (PCL). Among these, composite systems such as GelMA/alginate and PCL/GelMA are particularly widely applied, as they combine favorable biological performance with enhanced structural integrity and tunable mechanical properties. Such hybrid bioinks are especially important for replicating the load-bearing environment of the mandibular condyle [46,47,48].

Osteochondral Bioprinting

A key characteristic of condylar cartilage is its intimate structural and functional integration with the underlying subchondral bone. Consequently, contemporary research has increasingly focused on the development of osteochondral bioprinted constructs that reproduce the hierarchical organization of the native tissue, including a superficial fibrocartilaginous layer, a transitional interface zone, and a mineralized subchondral bone compartment. These constructs are typically fabricated using spatially controlled deposition of distinct cell populations and biomaterials within each region, enabling the recreation of the native tissue gradient. Preclinical studies in animal models have demonstrated promising results in terms of tissue integration, extracellular matrix deposition, and partial restoration of joint function [49,50,51].

Growth Factors

The induction and maintenance of chondrogenesis within bioprinted constructs rely heavily on the controlled delivery of bioactive signaling molecules. Key growth factors include transforming growth factor-β (TGF-β), bone morphogenetic proteins (BMP-2 and BMP-7), insulin-like growth factor-1 (IGF-1), and fibroblast growth factors (FGFs). Among these, TGF-β3 is particularly recognized for its strong chondrogenic and fibrochondrogenic induction potential, making it highly relevant for engineering fibrocartilage-like tissues characteristic of the temporomandibular joint. The spatial and temporal regulation of these growth factors within bioprinted scaffolds is considered essential for achieving stable and functional cartilage regeneration [52,53,54].

### 3.2. Bioprinting of the Subchondral Bone

The subchondral bone is a highly specialized osseous tissue located immediately beneath the articular cartilage of the mandibular condyle. It plays a crucial biomechanical and biological role by providing structural support to the overlying fibrocartilage, contributing to the dissipation and distribution of functional loads, and participating in the maintenance of overall joint homeostasis. Within the TMJ, the subchondral bone is continuously exposed to complex mechanical stimuli, including compressive, tensile, and shear forces generated during mastication, speech, and swallowing, rendering it particularly susceptible to degenerative and traumatic alterations [55,56].

In recent years, increasing evidence has indicated that pathological changes in the subchondral bone may precede or accompany the onset and progression of temporomandibular joint osteoarthritis. Early disease stages are characterized by trabecular bone remodeling, subchondral sclerosis, cyst formation, and erosive lesions, all of which directly influence the mechanical integrity and metabolic activity of the overlying articular cartilage. These findings have led to the recognition of the subchondral bone as a key therapeutic target in contemporary tissue engineering and regenerative medicine approaches for TMJ disorders [15,17].

Traditional reconstruction of mandibular condylar bone defects relies on autologous bone grafting, bone substitutes, and alloplastic implants. Although these techniques are widely used in clinical practice, they remain associated with significant limitations, including donor-site morbidity, restricted availability of autologous graft material, graft resorption, and suboptimal integration with surrounding native tissues. In addition, long-term functional outcomes may be compromised by mechanical mismatch between graft and host bone [57,58].

Cellular Sources

The selection of appropriate cellular sources is a critical determinant of success in subchondral bone bioprinting. The most commonly utilized cell types include BM-MSCs, AD-MSCs, periosteal stem cells, iPSCs, osteoblasts, and osteoprogenitor cells. Among these, mesenchymal stem cells are particularly advantageous due to their high proliferative capacity and their well-documented ability to undergo osteogenic differentiation and synthesize mineralized extracellular matrix under the influence of appropriate biochemical and mechanical cues. This makes MSCs a cornerstone cell population in bone tissue engineering strategies for TMJ reconstruction [59,60,61].

Biomaterials for Bone Bioprinting

Biomaterials used for subchondral bone bioprinting must meet several essential requirements, including biocompatibility, osteoconductivity, mechanical stability, and controlled biodegradation kinetics. These materials are typically categorized into bioceramics, synthetic polymers, and hydrogels. Bioceramics such as hydroxyapatite (HA), β-tricalcium phosphate (β-TCP), bioactive glass, and calcium phosphate cements closely resemble the inorganic phase of native bone and promote osteointegration and mineral deposition. Synthetic polymers, including polycaprolactone (PCL), PLA, and poly(lactic-co-glycolic acid) (PLGA), provide structural integrity and tunable degradation profiles. Among these, PCL is particularly widely used in TMJ-related bioprinting due to its excellent mechanical strength, processability, and long-term stability. Hydrogels such as GelMA, collagen, alginate, and hyaluronic acid provide a hydrated microenvironment that supports cell viability, proliferation, and differentiation, making them essential components of composite bioinks [62,63,64].

Osteogenic Growth Factors

Effective bone regeneration within bioprinted constructs relies heavily on the incorporation of osteoinductive signaling molecules. Key growth factors include bone morphogenetic proteins (BMP-2 and BMP-7), vascular endothelial growth factor (VEGF), platelet-derived growth factor (PDGF), and fibroblast growth factors (FGFs). Among these, BMP-2 is recognized as one of the most potent inducers of osteogenic differentiation, promoting mesenchymal stem cell commitment toward osteoblastic lineages and enhancing mineralized matrix formation in both in vitro and in vivo models [65,66,67].

Vascularization of Subchondral Bone Constructs

One of the most critical challenges in bone bioprinting is the establishment of a functional vascular network capable of sustaining cell viability and supporting long-term tissue integration. Without adequate vascularization, diffusion limitations severely restrict oxygen and nutrient transport, particularly in larger engineered constructs. To address this limitation, current strategies focus on the co-culture of osteogenic and endothelial cells, the incorporation of angiogenic biomaterials, and the design of pre-vascularized microchannel networks within bioprinted scaffolds. Additionally, the controlled and localized release of VEGF has been widely investigated to enhance angiogenesis and promote rapid vascular infiltration following implantation. These approaches are considered essential for achieving clinically viable subchondral bone regeneration in TMJ tissue engineering [68,69,70].

### 3.3. Bioprinting of the Articular Disc

The articular disc of the TMJ is a biconcave fibrocartilaginous structure located between the mandibular condyle and the articular eminence of the temporal bone. It plays a fundamental role in normal joint function by facilitating load distribution, maintaining joint congruency, absorbing mechanical shock, and enabling coordinated rotational and translational mandibular movements. Unlike hyaline cartilage present in most synovial joints, the TMJ disc is primarily composed of dense fibrocartilaginous connective tissue rich in type I collagen, with lower amounts of type II collagen and proteoglycans, which reflects its adaptation to tensile-dominant mechanical loading conditions [35,71,72].

Due to the absence of vascularization in its central region and its low cellular density, the articular disc exhibits extremely limited intrinsic regenerative capacity. Consequently, degenerative changes, perforations, and irreducible disc displacements are often associated with irreversible structural damage. These characteristics make the TMJ disc one of the primary targets for regenerative medicine approaches, particularly tissue engineering and 3D bioprinting strategies [35].

Degenerative disc pathology, internal derangements, and advanced disc displacement frequently result in persistent pain, functional impairment, and progressive joint degeneration. Conventional treatment modalities, including discoplasty, discectomy, and autologous grafting, have demonstrated variable long-term outcomes. Autografts, in particular, may undergo postoperative resorption, fibrotic remodeling, or structural deformation, limiting their ability to restore native disc function [73,74].

3D bioprinting offers a promising alternative by enabling the fabrication of patient-specific fibrocartilaginous constructs with controlled geometry and internal architecture. Through the precise spatial deposition of cells, biomaterials, and bioactive factors, it is possible to engineer constructs that mimic both the structural and functional properties of the native TMJ disc, including its anisotropic collagen organization and zonal mechanical behavior [35]. A major challenge in TMJ disc bioprinting lies in reproducing its highly heterogeneous internal organization. The disc is not a uniform structure but exhibits distinct regional variations in fiber orientation and mechanical properties. The central zone is thinner and primarily subjected to compressive forces, whereas the peripheral regions contain densely organized collagen fibers oriented to resist tensile loading. The intermediate zone represents a transitional fibrocartilaginous region that contributes to stress distribution and overall biomechanical stability. This structural complexity necessitates the development of multilayered and spatially controlled bioprinted constructs capable of replicating native tissue gradients. By modulating cell density, scaffold composition, and bioink properties across different layers, it is possible to engineer functional gradients that approximate the native disc architecture [71,72].

Cellular Sources

Successful bioprinting of the TMJ articular disc depends critically on the selection of appropriate cell populations with the capacity for fibrocartilaginous differentiation. The most commonly investigated cellular sources include BM-MSCs, AD-MSCs, synovium-derived mesenchymal stem cells (SM-MSCs), fibrochondrocytes, and iPSCs. Among these, mesenchymal stem cells are of particular interest due to their high plasticity and their ability to synthesize both type I and type II collagen under appropriate biochemical and mechanical stimulation, making them suitable for the regeneration of fibrocartilaginous tissues characteristic of the TMJ disc [59,75,76].

Biomaterials and Bioinks

Biomaterials used for articular disc bioprinting must ensure high cell viability, appropriate viscoelastic behavior, and sufficient mechanical resistance to repetitive functional loading. Natural biomaterials commonly applied include collagen, GelMA, alginate, fibrin, hyaluronic acid, and decellularized extracellular matrix (dECM), all of which provide bioactive cues that support cell adhesion, proliferation, and matrix deposition. Synthetic polymers such as PCL, polyethylene glycol (PEG), polyurethanes, and poly(lactic-co-glycolic acid) (PLGA) are frequently used to enhance structural stability and long-term mechanical integrity. In particular, composite systems such as PCL/GelMA have demonstrated promising results by combining the mechanical strength of synthetic polymers with the biological functionality of hydrogels, thereby providing a favorable microenvironment for fibrocartilage formation [72,77,78].

Growth Factors

The induction of fibrocartilaginous tissue formation within bioprinted constructs is strongly dependent on the controlled delivery of bioactive signaling molecules. Key growth factors include transforming growth factor-β1 (TGF-β1), transforming growth factor-β3 (TGF-β3), connective tissue growth factor (CTGF), BMPs, and insulin-like growth factor-1 (IGF-1). Among these, the combined application of CTGF and TGF-β3 has been shown to synergistically enhance fibrocartilaginous matrix formation and promote zonal organization resembling the native TMJ disc structure. The spatial and temporal regulation of these factors within bioprinted constructs is considered essential for achieving stable and functional fibrocartilage regeneration [71,78,79].

### 3.4. Bioprinting of Osteochondral Structures

Osteochondral constructs are complex bioengineered systems designed to simultaneously regenerate the articular cartilage and the underlying subchondral bone. In the context of the TMJ, this approach is of particular importance because the structural integrity and biomechanical function of the joint depend on the intimate interaction between the condylar fibrocartilage and the underlying subchondral bone. Damage to either component disrupts load transmission across the joint, leading to altered biomechanics, progressive cartilage degeneration, subchondral bone remodeling, and ultimately joint dysfunction [38,80,81].

The mandibular condyle is a highly specialized osteochondral structure composed of several distinct zones, including a superficial fibrous layer, a proliferative layer, a chondrogenic layer, a hypertrophic cartilage layer, and the underlying subchondral bone. Each of these regions exhibits a unique cellular composition, extracellular matrix organization, biochemical profile, and mechanical properties that collectively contribute to normal joint function. Consequently, successful regeneration of the TMJ requires not only the restoration of individual tissue components but also the accurate reproduction of their spatial organization and functional continuity [53].

The fundamental concept of osteochondral bioprinting is based on the fabrication of multiphasic constructs that reproduce the native tissue gradient. A typical osteochondral construct consists of three interconnected compartments: a superficial fibrocartilaginous layer that provides a low-friction articulating surface and resists compressive loading; an intermediate interface zone that facilitates mechanical and biological integration between cartilage and bone; and a mineralized subchondral bone layer that provides structural support, load transfer, and osseointegration. The incorporation of these gradient transitions enables more physiological distribution of mechanical stresses, improves integration between adjacent tissue compartments, and enhances the long-term stability of the engineered construct [24,82].

Cellular Sources

The successful fabrication of osteochondral constructs relies on the incorporation of distinct cell populations tailored to the biological requirements of each tissue compartment. For the cartilaginous region, the most commonly employed cell types include BM-MSCs, AD-MSCs, articular chondrocytes, and fibrochondrocytes. In contrast, the osseous compartment is typically populated with osteoblasts, osteoprogenitor cells, periosteal progenitor cells, or mesenchymal stem cells capable of osteogenic differentiation [83,84,85].

An increasingly attractive strategy involves the use of a single mesenchymal stem cell population throughout the entire construct. By exposing identical cells to compartment-specific biochemical and mechanical stimuli, localized differentiation into either chondrogenic or osteogenic phenotypes can be achieved. This approach simplifies construct fabrication, reduces manufacturing complexity, and facilitates regulatory standardization while maintaining the capacity to reproduce the heterogeneous cellular composition of the native osteochondral unit [38,53].

Biomaterials for Osteochondral Bioprinting

One of the principal advantages of three-dimensional (3D) bioprinting is the ability to fabricate multiphasic constructs using different biomaterials within a single integrated scaffold. This enables the recreation of the compositional and mechanical gradients characteristic of native osteochondral tissue [26].

Cartilaginous Component

The cartilaginous layer requires biomaterials that support chondrogenesis while maintaining high cell viability and extracellular matrix production. Frequently used bioinks include GelMA, type I and type II collagen, alginate, hyaluronic acid, and dECM. These biomaterials provide a hydrated, biologically active microenvironment that promotes cell attachment, proliferation, and synthesis of cartilage-specific matrix components, including collagen and glycosaminoglycans [17,82,86].

Osseous Component

The subchondral bone compartment requires materials with superior mechanical strength and osteoconductive properties. PCL, HA, β-tricalcium phosphate (β-TCP), bioactive glass, and calcium phosphate-based composites are among the most widely investigated biomaterials for this purpose. These materials provide structural stability, support mineral deposition, and facilitate osseointegration, making them particularly suitable for engineering the load-bearing region of the mandibular condyle [82,87].

Growth Factors and Biological Signaling

The spatially controlled delivery of growth factors is essential for directing lineage-specific differentiation within osteochondral constructs. Chondrogenic differentiation is primarily stimulated by transforming growth factor-β1 (TGF-β1), TGF-β3, and IGF-1, whereas osteogenesis is promoted by BMP-2 and BMP-7, VEGF, and PDGF. The incorporation of these signaling molecules into bioprinted scaffolds through controlled-release systems enables the establishment of localized biochemical gradients that closely mimic the molecular events occurring during embryonic osteochondral development and postnatal tissue regeneration [38,72,88].

Immunogenicity remains an important consideration in the development of biomaterials for TMJ bioprinting, as excessive host immune responses may impair tissue integration and compromise long-term construct performance. Consequently, current research increasingly focuses on biomaterials with high biocompatibility and low immunogenic potential. Naturally derived hydrogels, including collagen, GelMA, fibrin, and hyaluronic acid, are widely investigated because they provide a favorable microenvironment for cell survival while minimizing adverse inflammatory reactions. Decellularized extracellular matrix bioinks further reduce immunogenicity by removing cellular components while preserving tissue-specific extracellular matrix composition and biological signaling cues. In addition, surface modification of synthetic polymers and the incorporation of immunomodulatory biomolecules have shown promise in enhancing host integration and reducing foreign body responses. Although these strategies have demonstrated encouraging preclinical results, further studies are needed to evaluate their long-term immunological safety and clinical efficacy in TMJ reconstruction [85,89,90,91].

The combination of gradient biomaterials, compartment-specific cellular differentiation, and precisely regulated bioactive signaling is currently regarded as one of the most promising strategies for engineering functional osteochondral constructs capable of restoring the complex architecture and biomechanics of the temporomandibular joint.

**Figure 3:**
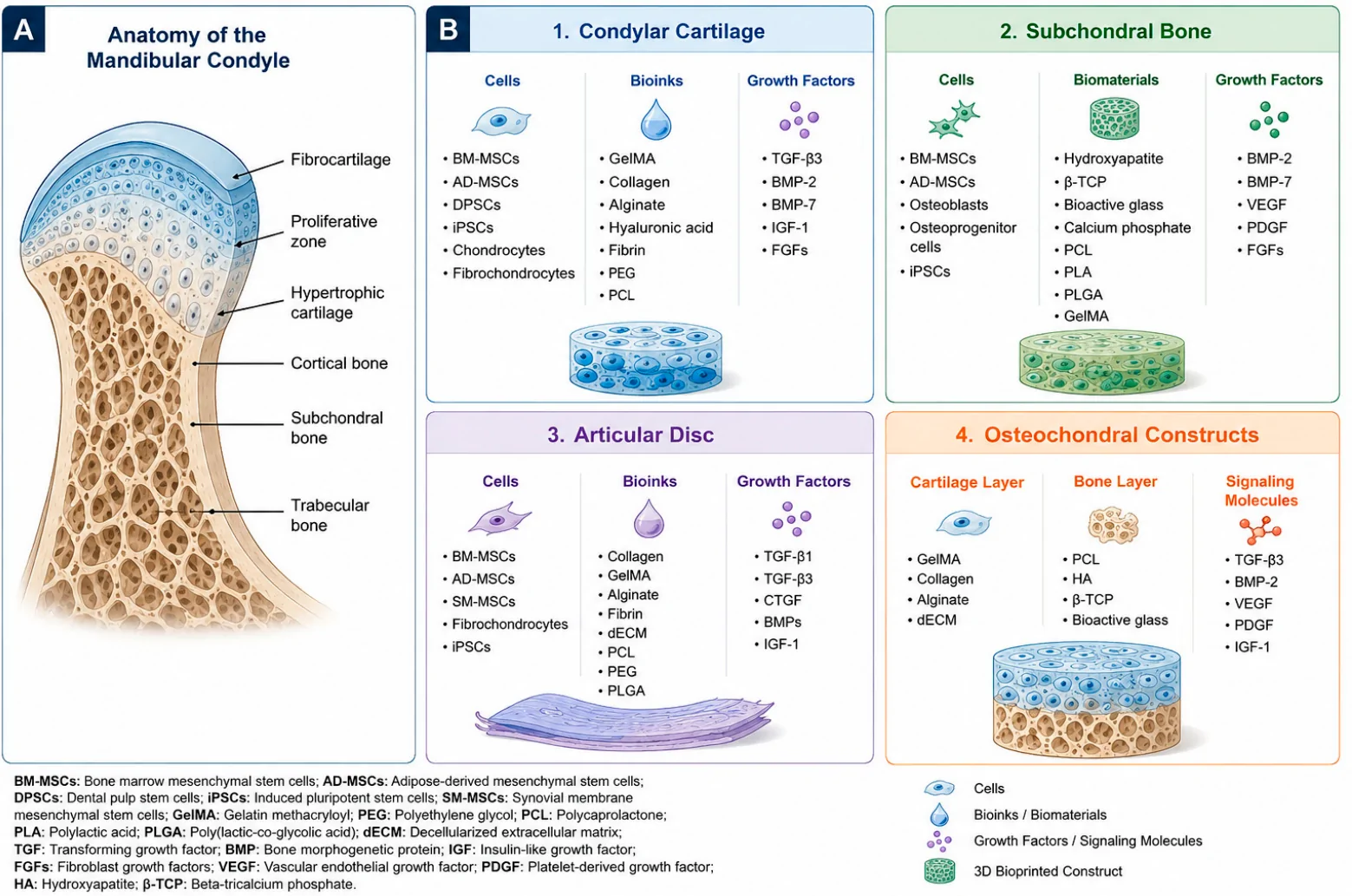
Tissue-specific components used in temporomandibular joint bioprinting. (**A**) Anatomical organization of the mandibular condyle showing the principal osteochondral tissues involved in regeneration. (**B**) Summary of the major cellular sources, biomaterials/bioinks, and growth factors investigated for the bioprinting of condylar cartilage, subchondral bone, articular disc, and integrated osteochondral constructs.

The information can be summarized in Table 3 and Figure 3:

## 4. Challenges

Despite remarkable advances in tissue engineering and 3D bioprinting, the development of a functional replacement for the TMJ remains one of the greatest challenges in regenerative medicine. Unlike most synovial joints, the TMJ is a highly specialized biological system composed of multiple tissues with distinct embryological origins, structural organizations, biochemical compositions, and biomechanical properties. The simultaneous regeneration of the condylar fibrocartilage, subchondral bone, and articular disc therefore presents substantial scientific, technological, and clinical challenges for the development of functional bioprinted constructs.

### 4.1. Challenges in Condylar Fibrocartilage Regeneration

Regeneration of the mandibular condylar fibrocartilage represents one of the greatest obstacles in TMJ tissue engineering because this tissue differs fundamentally from hyaline cartilage. The condyle consists predominantly of fibrocartilage rich in type I collagen, exhibits limited intrinsic healing capacity, and is continuously exposed to complex compressive, tensile, and shear forces generated during mastication and mandibular movement. Therefore, bioprinted constructs intended for osteoarthritis, progressive condylar resorption, or traumatic condylar defects must simultaneously provide sufficient mechanical strength while maintaining high cellular viability and supporting long-term chondrogenic differentiation. Achieving these competing biological and mechanical requirements remains a major challenge for clinical translation [37,38,92].

### 4.2. Challenges in Subchondral Bone Regeneration

Subchondral bone regeneration is particularly important for the treatment of condylar resorption, extensive traumatic defects, and post-oncologic reconstruction. The principal limitation is the establishment of a functional vascular network capable of supplying oxygen and nutrients throughout large engineered constructs. Insufficient vascularization leads to poor cell survival, delayed osseointegration, and incomplete bone regeneration. Current approaches—including endothelial cell co-culture, angiogenic biomaterials, sacrificial bioinks, and microvascular channel fabrication—have shown encouraging preclinical results but remain insufficiently validated for routine clinical use. In addition, balancing scaffold degradation with the rate of new bone formation continues to represent a significant challenge [55,62,93].

### 4.3. Challenges in Articular Disc Engineering

Engineering the TMJ articular disc remains particularly demanding because of its highly organized fibrocartilaginous architecture, regional variations in collagen fiber orientation, and limited vascularity. Degenerative disc disease, perforation, and irreducible disc displacement require constructs capable of reproducing both the anisotropic structure and the complex viscoelastic behavior of the native disc. Although multilayer hydrogels and composite scaffolds have demonstrated promising experimental performance, reproducing the zonal organization and long-term functional durability of the articular disc under physiological loading remains unresolved [71,72,94].

### 4.4. Appropriate Mechanical Properties

The TMJ is subjected to continuous cyclic loading during mastication, speech, and swallowing. Functional loading may reach several hundred newtons and is repeated thousands of times each day. Most hydrogel-based bioinks provide excellent cytocompatibility and support cell viability but possess inadequate mechanical strength for load-bearing applications. Conversely, synthetic polymers offer superior mechanical performance but often lack the biological properties required to promote cell attachment, proliferation, and tissue regeneration. Achieving an optimal balance between biomechanical stability and biological functionality therefore remains one of the principal challenges in TMJ bioprinting [24,54].

### 4.5. Vascularization of the Subchondral Bone

Successful regeneration of the osseous component depends on the establishment of a functional vascular network. Without sufficient vascularization, cells located within the interior of large bioprinted constructs are deprived of oxygen and nutrients, resulting in hypoxia, cell death, and ultimately implant failure. Consequently, the development of pre-vascularized constructs incorporating endothelial cells, angiogenic biomaterials, or bioprinted microvascular channel networks has become one of the most active areas of research in bone tissue engineering [84,88,95].

### 4.6. Integration Between Tissue Compartments

The native TMJ contains highly specialized transition zones between fibrocartilage and subchondral bone, as well as between the articular disc and adjacent articulating surfaces. These interfaces ensure efficient load transfer while preserving the structural and functional integrity of the joint. Reproducing these complex osteochondral interfaces remains a major challenge in bioprinting. Inadequate integration between adjacent tissue compartments may result in delamination, mechanical instability, impaired load transmission, and premature construct failure [17,88].

### 4.7. Selection of Appropriate Cell Sources

The cellular component is fundamental to the success of any bioprinted construct. One of the greatest challenges is directing cell differentiation toward the formation of fibrocartilage, which is structurally and biologically distinct from the hyaline cartilage found in most synovial joints. Although mesenchymal stem cells remain the most widely investigated cell source, important questions remain regarding the precise regulation of lineage-specific differentiation, long-term cell survival, phenotypic stability, and the prevention of undesirable hypertrophic differentiation or ectopic ossification [84,96].

### 4.8. Controlled Biomaterial Degradation

The degradation kinetics of scaffold materials must be carefully synchronized with the rate of new tissue formation. Excessively rapid degradation compromises mechanical integrity before sufficient extracellular matrix has been deposited, whereas excessively slow degradation may impede tissue remodeling and integration with host tissues. Optimizing this balance remains a critical objective in the design of biomaterials for osteochondral bioprinting [17,97].

### 4.9. Long-Term Functional Stability

Most preclinical studies evaluate engineered constructs over relatively short observation periods, typically ranging from several weeks to a few months after implantation. Consequently, there is limited evidence regarding long-term biomechanical performance, resistance to repetitive functional loading, tissue remodeling, and durability under physiological conditions. These considerations are particularly important for the TMJ, which functions continuously throughout life and is exposed to millions of loading cycles associated with mastication, speech, and swallowing [84,88].

### 4.10. Manufacturing and Regulatory Challenges

The clinical translation of TMJ bioprinting is further constrained by significant manufacturing and regulatory challenges. These include the standardization of bioink formulations, reproducibility of the bioprinting process, large-scale manufacturing under Good Manufacturing Practice (GMP) conditions, and rigorous quality control of cell-based products. In addition, regulatory pathways for living bioprinted tissue constructs remain incompletely defined in many jurisdictions, creating substantial barriers to clinical approval and widespread implementation [98,99].

### 4.11. Overall Challenge

Future progress in TMJ bioprinting will depend on the successful integration of tissue-specific biomaterials, optimized cellular sources, controlled vascularization, biomechanically functional osteochondral interfaces, and standardized manufacturing protocols. Addressing these challenges will require close collaboration among clinicians, biomaterials scientists, cell biologists, bioengineers, and regulatory specialists to facilitate the transition from experimental research to safe and effective patient-specific therapies.

## 5. Emerging Trends and Future Perspectives

Recent advances in biomaterials, stem cell biology, computational modeling, and artificial intelligence (AI) are rapidly transforming the field of 3D bioprinting. These technological developments are expected to significantly expand the role of bioprinting in TMJ reconstruction and regenerative medicine, bringing the field closer to clinically applicable, patient-specific therapies. To provide a balanced perspective, the emerging technologies are discussed below according to their current level of scientific evidence and translational readiness.

### 5.1. Technologies Supported by Established Preclinical Evidence

Multimaterial Bioprinting

One of the most significant advances in tissue engineering is the development of multimaterial bioprinting, which enables the simultaneous deposition of multiple bioinks with distinct biological and mechanical properties within a single construct. This approach facilitates the fabrication of complex osteochondral tissues that closely replicate the hierarchical organization of the native TMJ, including the fibrocartilaginous articular surface, the osteochondral interface, and the subchondral bone. Compared with conventional single-material constructs, multimaterial bioprinting offers superior control over tissue composition, mechanical gradients, and biological functionality, thereby enhancing structural integration and regenerative potential [96,100,101].

Bioprinting with Decellularized Extracellular Matrix

The incorporation of dECM into bioinks has emerged as a promising strategy for enhancing the biological performance of engineered tissues. Decellularization removes cellular components while preserving the native extracellular matrix architecture, biochemical composition, and tissue-specific signaling molecules. As a result, dECM-based bioinks provide excellent biocompatibility, promote cell adhesion and proliferation, support lineage-specific differentiation, and more accurately reproduce the native tissue microenvironment. Tissue-specific dECM bioinks derived from condylar cartilage, subchondral bone, or fibrocartilage are currently being investigated to improve the regeneration of individual TMJ components and facilitate patient-specific tissue engineering [102,103].

Bioreactor Technologies and Functional Tissue Maturation

Bioreactors play an essential role in the maturation of engineered tissues prior to implantation. By providing tightly controlled biochemical and biomechanical environments, they promote chondrogenesis, osteogenesis, extracellular matrix deposition, angiogenesis, and tissue remodeling. For TMJ applications, dynamic bioreactors capable of applying cyclic compressive loading and multidirectional mechanical stimulation are particularly important, as they replicate the physiological loading conditions experienced during mastication and mandibular movement. Such mechanical conditioning has been shown to improve both the structural organization and functional properties of engineered osteochondral tissues [97,98].

### 5.2. Promising Technologies Under Active Development

Personalized Bioprinting

Personalized medicine has become a central paradigm in modern healthcare, and TMJ bioprinting is no exception. High-resolution imaging modalities, including CT, CBCT, and MRI, enable the generation of accurate three-dimensional digital models from which patient-specific implants can be designed and fabricated. Such individualized constructs are particularly valuable for reconstructing complex defects associated with condylar resorption, ankylosis, traumatic injuries, congenital anomalies, and post-oncological resections, where restoration of patient-specific anatomy is essential for optimal functional outcomes [102,104].

Vascularized Bioprinted Constructs

The generation of functional vascular networks remains one of the principal challenges in tissue engineering. Current research focuses on the development of pre-vascularized bioprinted constructs through the incorporation of endothelial cells, angiogenic growth factors, microfluidic technologies, and sacrificial bioinks that create perfusable vascular channels. These strategies aim to enhance oxygen and nutrient transport following implantation, thereby improving cell survival, accelerating tissue integration, and supporting the regeneration of large osteochondral constructs [70,71,96].

### 5.3. Emerging Future Technologies

Four-Dimensional (4D) Bioprinting and Smart Biomaterials

The next generation of biofabrication technologies is represented by 4D bioprinting, which combines additive manufacturing with stimuli-responsive (“smart”) biomaterials capable of changing their shape, mechanical properties, or biological behavior over time. These dynamic transformations can be triggered by external stimuli such as temperature, pH, mechanical loading, electromagnetic fields, or biochemical signals. In the context of TMJ reconstruction, 4D-bioprinted constructs may adapt to the evolving biomechanical environment following implantation, thereby promoting tissue remodeling, functional maturation, and long-term integration with host tissues [105,106,107,108].

Artificial Intelligence and Computational Modeling

AI and digital twin technologies are emerging as transformative tools in TMJ tissue engineering and 3D bioprinting. AI-driven machine learning algorithms can analyze large biological and biomechanical datasets to optimize scaffold architecture, internal porosity, biomaterial distribution, printing parameters, cellular organization, and growth factor delivery, thereby improving construct functionality, reproducibility, and manufacturing efficiency. In addition, AI models may predict cell differentiation, tissue maturation, and the mechanical performance of bioprinted constructs, facilitating the development of more reliable patient-specific therapies [109,110]. Digital twins, defined as virtual patient-specific replicas generated from imaging and clinical data, enable simulation of TMJ biomechanics, implant behavior, and tissue regeneration under physiological loading conditions before fabrication and implantation. These computational models support optimization of implant design, prediction of long-term functional outcomes, and personalized treatment planning while reducing experimental iterations, development time, and associated costs. Although AI-assisted bioprinting and digital twin technologies remain in the early stages of application for TMJ reconstruction, they represent promising strategies for accelerating the clinical translation of personalized regenerative therapies. [111,112].

Entire Temporomandibular Joint Bioprinting

The ultimate objective of TMJ tissue engineering is the fabrication of a fully functional bioengineered temporomandibular joint capable of replacing all major anatomical components, including the mandibular condyle, articular disc, and glenoid fossa. Although this concept remains at the experimental stage, rapid advances in multimaterial bioprinting, stem cell biology, organoid technology, and developmental bioengineering are progressively bringing this vision closer to reality. Future success will depend on the integration of advanced biomaterials, vascularization strategies, bioreactor maturation, and computational design to produce living joint replacements with long-term structural and functional stability [97,98,113].

Future Perspective

Overall, TMJ bioprinting is progressing from proof-of-concept studies toward increasingly sophisticated regenerative strategies. Technologies such as multimaterial bioprinting, advanced bioinks, and bioreactor-assisted tissue maturation are supported by substantial preclinical evidence, whereas AI-assisted design, 4D bioprinting, and complete joint fabrication remain promising future directions. Continued interdisciplinary collaboration among biomaterials scientists, bioengineers, clinicians, and computational researchers will be essential to bridge the gap between laboratory innovation and clinical application.

## 6. Conclusions

3D bioprinting has emerged as one of the most promising approaches for the regeneration of the TMJ, offering the potential to overcome many of the limitations associated with conventional reconstructive strategies. Advances in biomaterials, stem cell biology, multimaterial bioprinting, computational modeling, and artificial intelligence have significantly expanded the possibilities for engineering patient-specific osteochondral constructs that more closely replicate the complex anatomy, hierarchical organization, and biomechanical function of the native joint.

Despite these advances, several major challenges remain before TMJ bioprinting can achieve routine clinical application. These include the development of biomaterials with appropriate mechanical and biological properties, reliable vascularization of large tissue constructs, precise recreation of osteochondral interfaces, long-term functional stability under physiological loading, and the establishment of standardized manufacturing protocols and regulatory pathways. Addressing these challenges will require close collaboration among clinicians, biomaterials scientists, bioengineers, cell biologists, and computational researchers.

Future progress is expected to be driven by the integration of multimaterial bioinks, stimuli-responsive 4D biomaterials, tissue-specific decellularized extracellular matrix, advanced bioreactor technologies, patient-specific digital design, and artificial intelligence-assisted optimization. Together, these innovations have the potential to enable the fabrication of biologically functional, personalized TMJ replacements capable of restoring both anatomical integrity and physiological joint function.

Within the next 5–10 years, partial reconstruction of individual TMJ components, particularly osteochondral condylar defects and articular disc replacements, appears to be the most realistic clinical application of 3D bioprinting. Complete bioprinted TMJ replacement will require further advances in vascularization, biomaterial optimization, interface engineering, and regulatory approval. Although the clinical translation of whole-joint bioprinting remains at an early stage, the rapid pace of innovation in regenerative medicine suggests that bioengineered TMJ reconstruction is becoming an increasingly realistic therapeutic strategy. While the fabrication of a fully functional bioprinted temporomandibular joint will require further multidisciplinary research and long-term clinical validation, current experimental evidence indicates that this objective is no longer merely theoretical but represents a realistic direction for the future of craniofacial regenerative medicine.

## Figures and Tables

**Table 1:** Classification of the TMJ disorders that may benefit from tissue engineering and 3D bioprinting.

Disease/Clinical Condition	Pathology	Primary Defect Type	Target Tissue for Regeneration	Clinical Reconstruction Need	Potential Role of 3D Bioprinting
**Temporo-mandibular Joint Osteoarthritis**	Degenerative	Progressive fibrocartilage degeneration with subchondral bone remodeling	Condylar fibrocartilage and subchondral bone (osteochondral unit)	Restoration of joint biomechanics, pain reduction, prevention of further degeneration	Patient-specific osteochondral constructs combining cartilage and bone regeneration
**Severe Condylar Resorption**	Degenerative/Idiopathic	Irreversible loss of condylar cartilage and bone	Condyle (cartilage + subchondral bone)	Anatomical reconstruction of the mandibular condyle and restoration of occlusion	Customized osteochondral bioprinted condylar replacement
**Traumatic Condylar Defects**	Traumatic	Partial or complete condylar tissue loss	Bone and fibrocartilage	Reconstruction of post-traumatic defects and recovery of joint function	Patient-specific bioengineered osteochondral implants
**Temporo-mandibular Joint Ankylosis**	Post-traumatic/Inflammatory	Fibrous or bony fusion of the joint	Entire osteochondral unit	Restoration of mandibular mobility and prevention of re-ankylosis	Biological replacement of damaged joint components following ankylosis release
**Degeneration and Perforation of the Articular Disc**	Degenerative/Mechanical	Fibrocartilaginous disc damage	Articular disc	Restoration of load distribution, shock absorption, and joint stability	Fibrocartilage bioprinting using multilayer scaffolds and hydrogels
**Congenital and Acquired Condylar Defects**	Developmental	Partial or complete absence of the condyle	Condyle and osteochondral tissues	Anatomical reconstruction and restoration of facial growth and function	Personalized bioengineered condylar replacement (future application)
**Post-resection Defects Following Tumor Surgery**	Oncologic	Composite defects involving bone, cartilage, and surrounding tissues	Osteochondral complex ± adjacent soft tissues	Reconstruction of extensive craniofacial defects with patient-specific implants	Image-guided CAD/CAM-assisted bioprinting of customized constructs

**Table 2:** Keyword combination and filters during the search process.

Keyword Combinations	Filters Applied	Primary Purpose
(“temporomandibular joint” OR TMJ) AND (“3D bioprinting” OR bioprinting OR biofabrication)	English; 2010–2026; journal articles and reviews	Identification of original research and review articles on TMJ bioprinting
(“TMJ” OR “temporomandibular joint”) AND (“biomaterials” OR bioinks OR scaffolds)	English; 2010–2026; journal articles and reviews	Identification of biomaterial and bioengineering studies
“temporomandibular joint” + bioprinting + cartilage + bone + disc	English; 2010–2026; journal articles and reviews	Identification of the properties of each tissue
TMJ + bioprinting + Challenges + Emerging Trends + Future Perspectives	English; 2010–2026; journal articles and reviews	Identification of Challenges, Emerging Trends and Future Prospects for Bioprinting of TMJ

**Table 3:** Relationship Between TMJ Clinical Indications, Target Tissues, Biomaterials, Bioinks/Scaffolds, and Current Level of Evidence.

Clinical Indication/Defect	Target Tissue	Principal Biomaterials	Representative Bioinks/Scaffolds	Main Cellular Sources	Current Level of Evidence
**TMJ osteoarthritis**	Condylar fibrocartilage + subchondral bone	GelMA, collagen, alginate, hyaluronic acid, PCL, hydroxyapatite (HA), β-TCP	GelMA/alginate hydrogels, PCL–GelMA composite scaffolds, osteochondral multilayer constructs	BM-MSCs, AD-MSCs, chondrocytes	In vitro, small-animal and large-animal preclinical studies
**Progressive condylar resorption**	Osteochondral condyle	PCL, HA, calcium phosphate ceramics, collagen	Patient-specific CAD/CAM osteochondral scaffolds	MSCs, osteoblasts	Preclinical; no routine clinical bioprinting yet
**Traumatic condylar defects**	Bone + fibrocartilage	PCL, PLA, PLGA, HA	Composite osteochondral scaffolds	BM-MSCs, osteoprogenitor cells	Preclinical proof-of-concept
**TMJ ankylosis**	Entire osteochondral unit	PCL, collagen, HA, bioactive ceramics	Customized multilayer osteochondral constructs	MSCs	Experimental and early translational research
**Articular disc degeneration/perforation**	Fibrocartilaginous disc	Collagen, GelMA, fibrin, alginate, dECM	Hydrogel-based multilayer scaffolds, PCL/GelMA composites	Fibrochondrocytes, BM-MSCs, AD-MSCs, iPSCs	Extensive in vitro and animal studies
**Congenital condylar defects**	Condyle (cartilage + bone)	PCL, HA, β-TCP	Patient-specific osteochondral scaffolds	MSCs	Conceptual and preclinical stage
**Post-oncologic mandibular condyle defects**	Composite bone/cartilage defect	PCL, calcium phosphate, bioactive ceramics	CAD/CAM-designed customized constructs	MSCs, osteoblasts	Experimental translational research

## Data Availability

No new data were created or analyzed in this study. Data sharing is not applicable to this article.

## Abbreviations

The following abbreviations are used in this manuscript:

TMJ	temporomandibular joint
TMDs	temporomandibular disorders
DC/TMD	Diagnostic Criteria for Temporomandibular Disorders
CAD	computer-assisted design
CT	computed tomography
CBCT	cone-beam computed tomography
MRI	magnetic resonance imaging
AI	artificial intelligence
MSCs	mesenchymal stem cells
BM-MSCs	bone marrow-derived mesenchymal stem cells
AD-MSCs	adipose-derived mesenchymal stem cells
SM-MSCs	synovium-derived mesenchymal stem cells
DPSCs	dental pulp stem cells
iPSCs	induced pluripotent stem cells
TGF-β	transforming growth factor-β
CTGF	connective tissue growth factor
BMP	bone morphogenetic proteins
IGF-1	insulin-like growth factor-1
FGFs	fibroblast growth factors
HA	hydroxyapatite
β-TCP	β-tricalcium phosphate
PCL	polycaprolactone
PLA	polylactic acid
PEG	polyethylene glycol
PLGA	polylactic-co-glycolic acid
GelMA	gelatin methacrylate
VEGF	vascular endothelial growth factor
PDGF	platelet-derived growth factor
dECM	decellularized extracellular matrix
